# Characterization of novel transforming growth factor-beta type I receptors found in malignant pleural effusion tumor cells

**DOI:** 10.1186/1471-2199-8-72

**Published:** 2007-08-17

**Authors:** Kuo-Li Chen, Wan-Hsin Liu, Yi-Yuan Yang, Sy-Jye C Leu, Neng-Yao Shih

**Affiliations:** 1 Graduate Institute of Life Sciences, National Defense Medical Center, Taipei 114, Taiwan; 2 National Institute of Cancer Research, National Health Research Institutes, Taipei 114, Taiwan; 3 School of Medical Laboratory Science and Biotechnology, Taipei Medical University, Taipei 110, Taiwan; 4 Graduate Institute of Cell and Molecular Biology, Taipei Medical University, Taipei 110, Taiwan

## Abstract

**Background:**

Tumors expressing a transforming growth factor-beta type I receptor (TβRI) mutant with sequence deletions in a nine-alanine (9A) stretch of the signal peptide are reported to be highly associated with disease progression. Expression of this mutant could interfere with endogenous TGFβ signaling in the cell. However, little is known about the importance of the remaining part of the signal peptide on the cellular function of TβRI.

**Results:**

We cloned and identified four new in-frame deletion variants of TβRI, designated DM1 to DM4, in pleural effusion-derived tumor cells. Intriguingly, DM1 and DM2, with a small region truncated in the putative signal peptide of TβRI, had a serious defect in their protein expression compared with that of the wild-type receptor. Using serial deletion mutagenesis, we characterized a region encoded by nucleotides 16–51 as a key element controlling TβRI protein expression. Consistently, both DM1 and DM2 have this peptide deleted. Experiments using cycloheximde and MG132 further confirmed its indispensable role for the protein stability of TβRI. In contrast, truncation of the 9A-stretch itself or a region downstream to the stretch barely affected TβRI expression. However, variants lacking a region C-terminal to the stretch completely lost their capability to conduct TGFβ-induced transcriptional activation. Intriguingly, expression of DM3 in a cell sensitive to TGFβ made it significantly refractory to TGFβ-mediated growth inhibition. The effect of DM3 was to ablate the apoptotic event induced by TGFβ.

**Conclusion:**

We identified four new transcript variants of TβRI in malignant effusion tumor cells and characterized two key elements controlling its protein stability and transcriptional activation. Expression of one of variants bestowed cancer cells with a growth advantage in the presence of TGFβ. These results highlight the potential roles of some naturally occurring TβRI variants on the promotion of tumor malignancy.

## Background

Malignant pleural effusions are good sources of transforming growth factor β (TGFβ)-resistant tumor cells. They frequently contain high levels of TGFβ of around 7.5 ng/mL [1]. However, epithelial tumor cells shedding or migrating from local primary tumor sites are still alive and grow well in the pleural effusions. Such cells are believed to have some defects in the TGFβ receptors or in receptor-mediated signaling pathways. Therefore, in the present study, tumor cells derived from pleural effusions of patients with lung cancer were chosen for examining the gene expression and gene integrity of these receptors.

Type I TGFβ receptor (TβRI) is a key signaling receptor for TGFβ to conduct Smad-mediated transcriptional activation in a cell. There are three major cell surface receptors characterized for TGFβ ligands, designated TβRI, TβRII, and TβRIII. In early signaling events, TGFβ binds to its cell surface TβRII, followed by the recruitment and phosphorylation of TβRI on a juxtamembrane glycine/serine (GS)-rich domain. This phosphorylation results in the association of R-Smads (Smad2 and Smad3) with a shared partner, Smad4, to form heterotrimers that undergo nuclear translocation to regulate TGFβ-responsive genes. The putative function of TβRIII in these events is to facilitate ligand binding to TβRI and TβRII [2]. Among these TGFβ receptors, the type I plays a pivotal role in conducting the TGFβ stimulus from cell surface into the nucleus to deliver various biological outcomes. Functional disruption, mutation, or aberrant expression of the TGFβ type I receptor gene (*Tgfbr1*) is frequently associated with human diseases, including cancers [3-6].

The *Tgfbr1 *gene is a tumor susceptible allele. Over 30% of patients with ovarian cancer harbor somatic changes in exons 1 to 6 [7-9]. Most changes are in-frame deletions or insertion mutations in the nine-alanine (9A) stretch of the putative signal peptide or missense mutations in the catalytic kinase domain of the receptor. Other TβRI transcript variants with sequence defect in the peptide have been also reported in the rat kidney [10]. Notably, a gene variant with a three-alanine deletion (*Tgfbr1-6A*) in the 9A stretch was found recently in a variety of cancers [5,7,11,12], suggesting that this signal peptide is a susceptible site for its gene mutation. Transfection of this variant has been shown to convert TGFβ growth inhibitory signals into growth stimulatory signals in cells [13]. However, little is known about cellular functions of regions in the signal peptide other than the 9A stretch.

We found four naturally occurring TβRI variants in three patients with lung cancer. All variants have different in-framed truncations in the signal peptide. Molecular dissection of the peptide using serial deletion mutagenesis characterized two novel functional regions that participate in maintaining TβRI protein stability and receptor-mediated transcriptional regulation in cells. Expression of one of these variants in a cell could lead to its refractoriness to TGFβ, supporting an idea that naturally occurring TβRI may have a role in tumor progression. Collectively, our data suggest that the signal peptide of TβRI can be a susceptible region for mutation resulting in TGFβ insensitivity in cancer. Mutations occurred in different regions of the signal peptide may display distinct TβRI defect modes and suggest that the variants with these mutations may represent potential therapeutic targets in cancers.

## Results

### Sequence deletions in the signal sequence of TβRI from effusion-derived tumor cells

**Figure 1:**
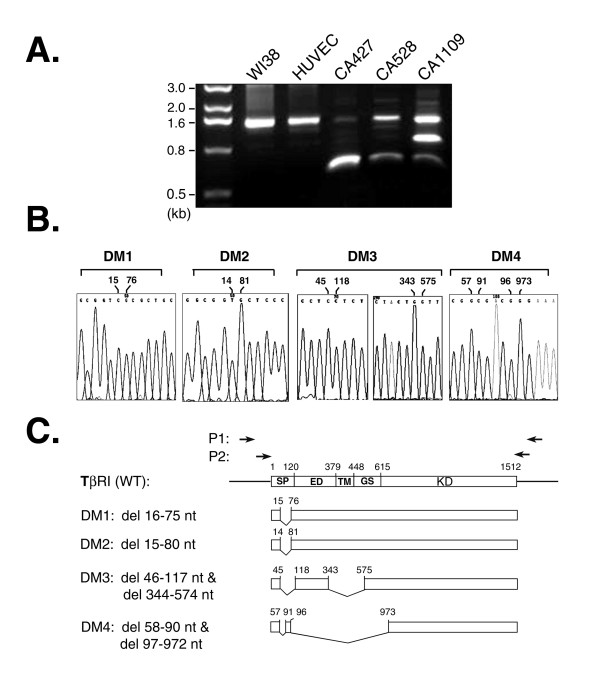
**The existence of *Tgfbr1 *transcript variants in pleural effusion-derived tumor cells**. *Panel A: RT-PCR*. Total RNA was extracted from WI38, HUVEC, and pleural effusion-derived tumor cells followed by reverse transcription. Nested PCR reactions amplified *Tgfbr1 *coding sequences using two pairs of primers, P1 and P2, as indicated at the top of *Panel C*. The resultant amplicons were resolved in a 1% agarose gel followed by gel purification, gene cloning, and direct sequencing. The nucleotide sequences flanking the truncated region(s) in each variant are marked at the top of *Panel B*. *Panel C: Schematic illustration of the Tgfbr1 variants*. DM1 and DM2 are in-frame variants lacking nucleotides 16–75 and 15–80, respectively, located in the signal sequence of the *Tgfbr1 *gene. DM3 and DM4 represent cytoplasmic variants lacking transmembrane and partial intracellular domains, as indicated.

Four new *Tgfbr1 *transcript variants were found in pleural effusion-derived tumor cells. Total RNA was extracted from patient cells CA427, CA528, CA1109, and from the normal control cells, WI38 and HUVEC, and reverse-transcribed into cDNA. Nested-PCR amplification reactions using two pairs of primers, P1 and P2, located in the 5' and 3' untranslated regions were performed to clone out the endogenous *Tgfbr1 *transcript variants from the cells. Gel electrophoresis of the amplicons revealed that at least two additional *Tgfbr1 *transcript variants in the patient cells. However, no extra band was detected in the normal control cells, except for the 1.5 kb band representing the wild-type *Tgfbrl *transcript (Fig. 1A). After gene cloning and sequencing, four new in-framed deletion variants were identified in the patients totally. The nucleotide sequences of each variant in the truncated region are shown in Figure 1B and listed in Table 1. They were designated as DM1 and DM2, with sizes around 1.5 kb, and DM3 and DM4, with 0.3 kb and 0.9 kb deletions, respectively (Fig. 1C). Intriguingly, all the variants had varied nucleotide deletions in a sequence coding for the putative signal peptide of TβRI. The proteins encoded by the DM3 and DM4 transcript variants also lack the transmembrane domain and either partial or entire extracellular and GS-rich regions, respectively. Thus, the data suggest that the insensitivity of effusion tumor cells to TGFβ may be caused by defects in the protein integrity of TβRI.

**Table 1:** Sequence deletions in *Tgfbr1 *variants with differential gel-mobility

**Sample**	**Fast**	**Medium**	**Slow**
WI38			WT (6)
HUVEC			WT (6)
CA427	Δ58–90/Δ97–972 (5)		WT (6)
CA528	Δ58–90/Δ97–972 (5)		WT (6)
CA1109	Δ58–90/Δ97–972 (5)	Δ46–117/Δ344–574 (5)	Δ16–75 (2) & Δ15–80 (4)

Δ, nucleotide deletion; WT, wild-type; (n), number of clone sequenced

### TβRI deletion variants are not contributed by somatic mutation

**Figure 2:**
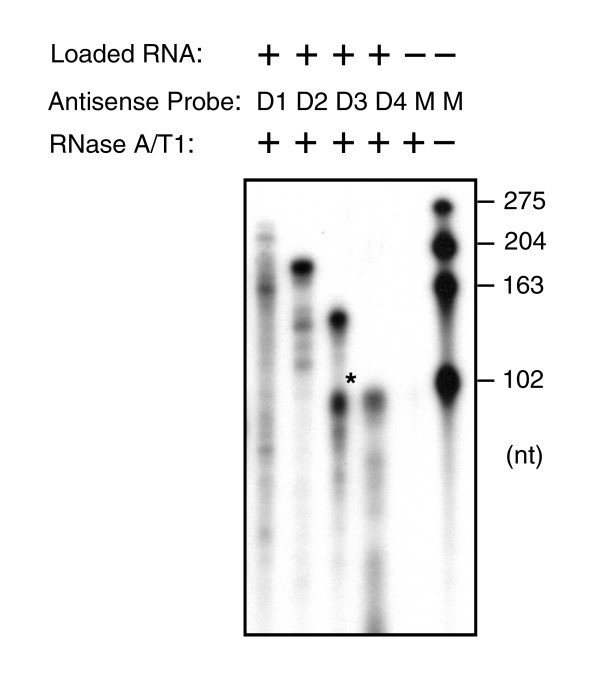
**Detection of transcripts of *Tgfbr1 *variants by RNase protection analysis**. Total RNA extracted from CA1109 effusion tumor cells were protected by corresponding antisense riboprobes, as indicated at the top. The riboprobes were synthesized by T7 RNA polymerase using T7 promoter-containing DNA fragment of individual variant as a template. The predicted protected fragments are 263 bp for D1 (DM1), 192 bp for D2 (DM2), 151 bp for D3 (DM3), and 90 bp for D4 (DM4). *Lanes 5 and 6: *the mixed riboprobes (M) were incubated with (+) or without (-) RNase A/T1, respectively. *Lanes 1–4: *the total RNA (10 μg aliquots) was hybridized with individual purified antisense riboprobes and the protected fragments were resolved on a 6% acrylamide/8 M urea sequencing gel. * Indicates an unidentified *Tgfbr1 *transcript variant in CA1109 tumor cells protected by the anti-DM3 riboprobe.

To detect the *in vivo *expression of *Tgfbr1 *transcript variants directly, ribonuclease protection assays were carried out using individual antisense riboprobes for these endogenous variants in CA1109 effusion cells. The data shown in Figure 2 confirm that the existence of these four transcript variants was not an artifact of the reverse transcription or PCR reactions but that they occurred naturally in the tumor cells. We subsequently genotyped *Tgfbr1 *gene from the tumor cells to determine if this deletion event was caused by a somatic mutation. Gene amplification was carried out by PCR amplification using published primers [9] to target the intronic sequences of exons 1, 2, or 3. The results obtained from direct sequencing showed no detectable mutations in the effusion tumor and normal control cells (data not shown). Apparently, the nucleotide deletions in the signal peptides sequence of those transcripts do not occur at the gene structural level but may be contributed by RNA editing or undefined post-transcriptional cellular processes.

### The N-terminal sequence of TβRI signal peptide controls its protein expression

**Figure 3:**
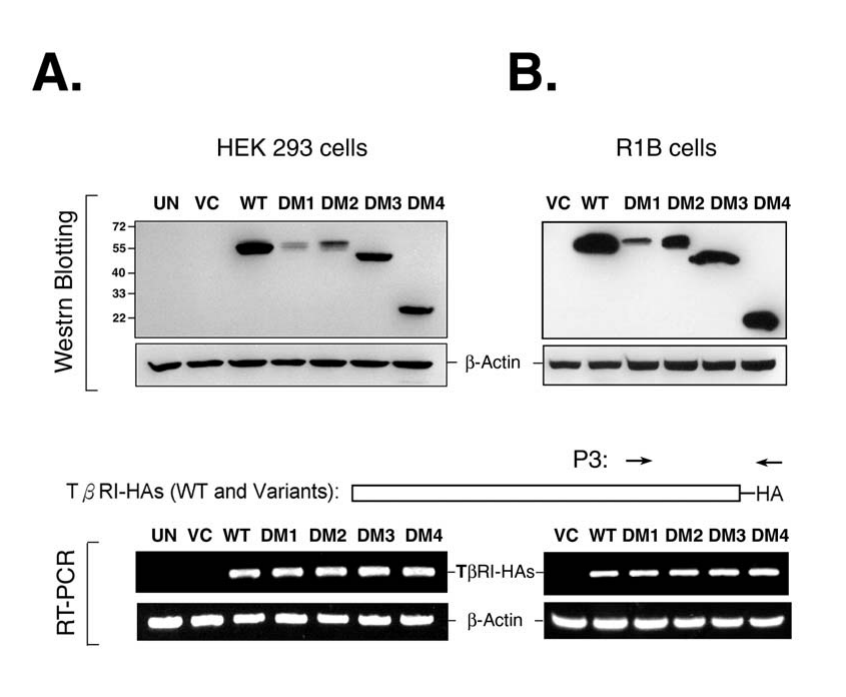
**Differential expression of TβRI variants**. HEK 293 or R1B cells were transiently transfected with a plasmid encoding for haemagglutinin (HA)-tagged wild-type TβRI (WT), DM1, DM2, DM3, DM4 or vector control (VC), as indicated at the top. Untransfected (UN) cells were also included as control. After transfection for 24 h, the cells were trypsinized and divided into two groups for either Western blotting or RT-PCR analyses of HEK293 transfectants in *Panel A *or R1B cells in *Panel B*. For Western blotting analyses, the exogenous WT TβRI and its variants were detected with an antibody specific to the HA tag. For PCR reactions, a pair of primers, P3, complementary to C-terminal and HA tag sequences of exogenous TβRI-HAs to determine the transcript levels of WT and its variants as indicated. A *β-Actin *transcript and its encoded protein were used as loading controls, as indicated in the middle.

Because no reliable antibody specific to TβRI was available, the coding sequences of these variants were constructed and expressed as C-terminal HA-tagged proteins in HEK293 and R1B cells. Western blotting analyses indicated that the variants had great differences in their protein expression. Notably, DM1, lacking nucleotides 16–75, and DM2, lacking nucleotides 15–80, were expressed at extremely low levels in the cells as compared with the expression of the wild-type control and the other two variants (Fig. 3A). The same expression pattern was also observed in R1B (Fig. 3A) and Cos-7 cells (data not shown), indicating that the differential expression must be cell type independent. An experiment using a plasmid encoding green fluorescence protein co-transfected with the individual variant genes showed that there were no substantial differences in gene transfection efficiency, ranging from 84.4% to 90.9% as determined by flow cytometric analyses. Consistently, reverse transcriptase (RT)-PCR analysis using P3 primers, as indicated in Figure 3, showed that no distinction in the level of RNA transcript among the exogenous variants was detected in the cells.

**Figure 4:**
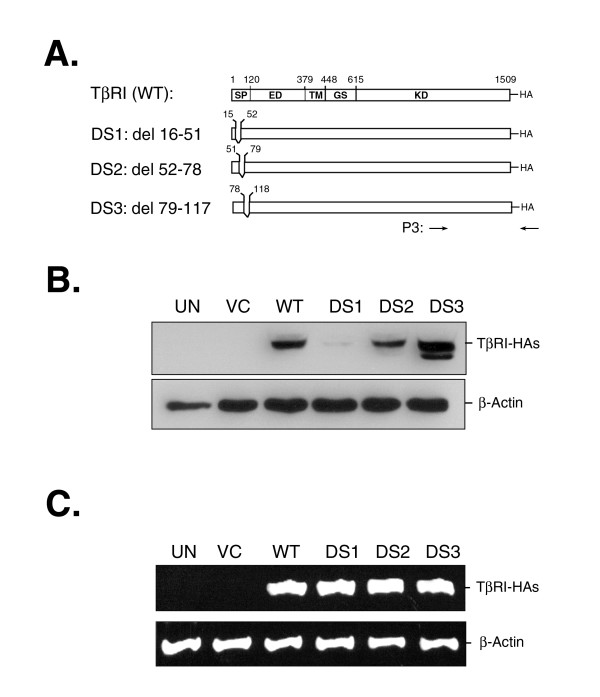
**Characterization of functional domains in the signal peptide of TβRI**. *Panel A: Schematic illustration of the TβRI mutants with deletions in the signal peptide*. Using wild-type *Tgfbr1 *(WT) as a template, the regions 5' or 3' to the 9A stretch sequence or the 9A stretch itself were truncated serially by site-directed deletion mutagenesis and designated as DS1, DS3, and DS2, respectively. HEK 293 cells were either untransfected (UN) or transfected with individual variants or vector control (VC) plasmid, as indicated at the top. The cells were divided into two groups for Western blotting or RT-PCR analyses 24 h after transfection. *Panel B: Western blotting*. Each lysate (30 μg) was resolved on a 10% SDS-polyacrylamide gel, immunoblotted with HA-specific antibody, and reprobed with anti-β-Actin antibody as a loading control. *Panel C: RT-PCR*. Total RNA was extracted from each transfectant and reverse transcribed. PCR reactions using P3 primers, as indicated at the bottom of *Panel A*, were utilized and the resultant amplicons were resolved on 1% agarose gels. A β-Actin transcript and its protein were used as loading controls, as indicated on the right.

To determine if an element in the signal peptide might affect TβRI expression, the sequence from nucleotide 16 to nucleotide 117 was serially deleted as shown schematically in Figure 4A. The DS1 mutant lacks nucleotides 16–51, which code for a peptide N-terminal to a 9A stretch. The DS2 mutant protein has a peptide truncation in the 9A stretch itself, which has been reported to be susceptible to nucleotide deletion in patients with several types of cancer [5,7,13]. The DS3 mutant, with deletion of nucleotides 79–117, encodes a protein lacking a peptide C-terminal to the 9A stretch. The data shown in Figure 4B clearly indicate that deletion of the small sequence of nucleotides 16–51 (DS1) is sufficient to impair the expression of TβRI protein. Accordingly, both DM1 and DM2 variants with deletion of this sequence also demonstrated the same defect in their protein expression. In Figure 4C, RT-PCR analysis further confirmed that differential protein expression among those deletion mutants was not because of differences in the levels of their transcripts. In contrast to DS1, the deletion of nucleotides 52–78 (DS2) or 79–117 (DS3) affected expression only slightly. Thus, the peptide encoded by nucleotides 16–51 is a key element modulating the cellular expression of the TβRI protein.

### Role of the signal peptide on protein stability of TβRI

**Figure 5:**
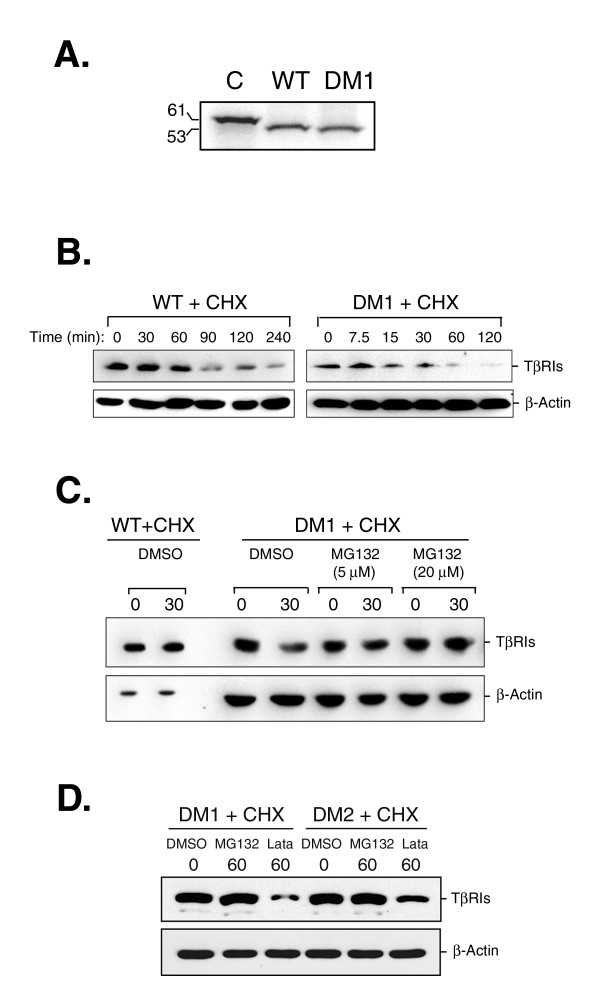
**Protein stability of exogenous wild-type TβRI-HA and the DM1 variant in HEK293 cells**. *Panel A: Translation efficiency*. A plasmid (1 μg) encoding for wild-type (WT) TβRI or DM1 was transcribed and translated *in vitro *using a coupled reticulocyte lysate system. ^35^S-labeled proteins were resolved on a 10% SDS-polyacrylamide gel and visualized by autoradiography. The control plasmid (C) encoded for luciferase polypeptide (61 KDa). *Panel B: Half-lives of wild-type TβRI and DM1*. Cells were transfected with a plasmid encoding for wild-type TβRI (WT) or the DM1 variant. After 24 h transfection, the transfectants were treated with 50 μg/mL cycloheximide (CHX) for the designed time courses, as indicated at the top. WT (15 μg) and DM1 (150 μg) transfectant lysates were analyzed by Western blotting using an anti-HA antibody. *Panel C & D: Protein stability of DM1 and DM2 associated with proteosome-related degradation*. Transfectants were treated simultaneously with CHX and 5 μM, 20 μM MG132, or DMSO (vehicle control) for 30 min in *Panel C*. Transfectants treated with CHX and 20 μM MG132 or 10 μM lactacystin (Lata) for 60 min were shown in *Panel D*. Protein levels were determined by Western blotting of WT, DM1, and DM2 transfectant lysates using an anti-HA antibody. The same blot was reprobed with β-actin antibody as a control.

To determine if a sequence deletion of the signal peptide might affect translation efficiency resulting in failure of expression of TβRI, the DM1 was chosen because it had the lowest protein expression among those variants. The experiment coupling *in vitro *transcription and translation demonstrated no distinct difference between DM1 and the wild-type control on the translation efficiency (Fig. 5A).

To examine the possibility that a lack of the peptide encoded by nucleotides 16–75 would affect the turnover rate of the DM1 protein, its half-life and stability were examined. The DM1 gene was transfected into HEK 293 cells followed by blocking translation machinery with cycloheximide (CHX). The half-life of the DM1 protein (about 30 min) was much shorter than that of the wild-type protein (90 min) (Fig. 5B). These data are in agreement with a previous report using pulse-chase metabolic labeling [5]. Co-treatment with an ubiquitin-proteasome inhibitor, MG132, and cycloheximide rescued the DM1 protein from degradation (Fig. 5C and 5D). The same conclusion was obtained from an experiment using the DS1 variant as a target protein (data not shown). However, this effect was not seen in the experiment using a 20S proteasome inhibitor, lactacystin (Fig. 5D), indicating that degradation of the DM1 and DS1 proteins is through an ubiquitin-dependent process. Thus, the data clearly indicate that the peptide encoded by nucleotides 16–51 is a key element involved in TβRI protein stability.

### Functional activation of TβRI variants in R1B cells

**Figure 6:**
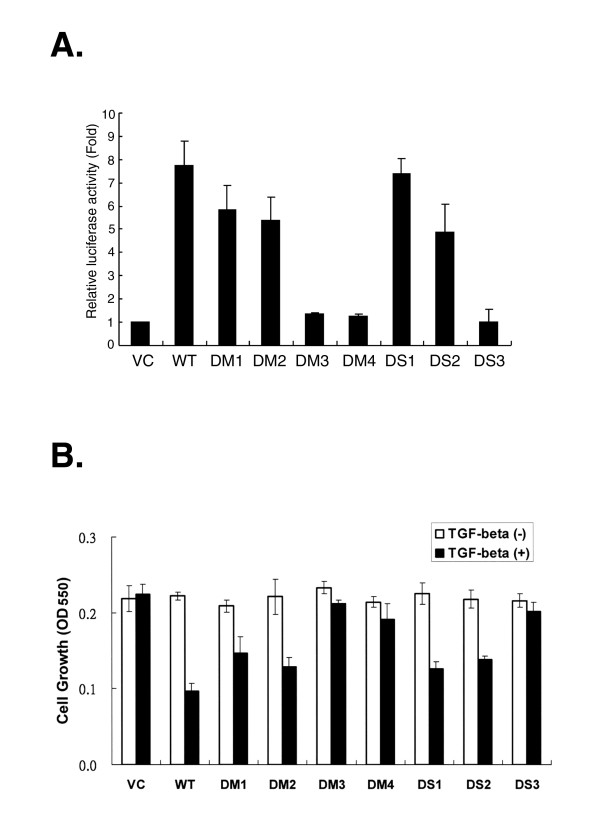
**Transcription activation induced by TβRI variants**. *Panel A. Transcriptional activation of TβRI variants*. R1B cells were transiently transfected with 1 μg of either wild-type (WT) TβRI or truncated variants, as indicated in the bottom, 1 μg of p3TP-Lux, and 0.05 μg of pRL-TK plasmid which was used to correct the differences in transfection efficiency between experiments. After transfection for 24 h, the cells were treated with TGFβ1 (5 ng/mL) for 24 h and luciferase activities in each lysates were determined using a dual-luciferase reporter assay system (Promega). Data are the mean ± SEM luciferase activities of four independent experiments normalized to individual protein level. *Panel B. Effects of TβRI variants on TGFβ-induced cell growth*. R1B cells were transfected with each variant gene as indicated. The cell growth rates of the transfectants in the presence or absence of TGFβ1 were determined using standard MTT assays as described in the "Methods" section. Data are presented as the mean ± SEM of OD_550 _absorbance values of three independent experiments.

To test function of these naturally occurring TβRI variants on TGFβ-mediated signaling, receptor-mediated transcriptional activation was examined by a reporter gene assay. The p3TP-Lux reporter containing a TPA-responsive element and *PAI-1 *promoter was co-transfected with individual variant genes into the TβRI-null cell line, R1B. In response to TGFβ stimulation, receptor-mediated transcriptional activation was determined from the corresponding luciferase activity. Compared with transcriptional activation mediated by wild-type TβRI after protein normalization, truncation of a peptide encoded by nucleotides 16–51 for the DS1 mutant did not affect its receptor function (Fig. 6A). Similar results were also seen in the DM1 and DM2 mutants with deletion of this peptide. In contrast, truncation of a peptide C-terminal to the 9A stretch (DS3) dramatically impaired its capability to conduct TGFβ signaling. A consistent result was also observed in the DM3 and DM4 variants lacking this peptide. Thus, these data suggest that at least two functional subdomains are located on the putative signal peptide for TβRI to control its protein expression and downstream signaling.

To examine if transcriptional activation induced by these variants might affect cellular function, R1B cells were transfected with individual variant genes and their growths were measured using a standard MTT assay. In the presence of TGFβ, the DM1, DM2, and DS1 variants significantly suppressed the growth of R1B cells although their protein expression levels were much lower than that of the wild-type one (Fig. 6B). This data strongly suggests that the expression levels are sufficient to transduct the extracellular TGFβ signal, activate gene transcription, and exert biological action in R1B cells. In contrast, lack of a peptide C-terminal to the 9A stretch in DM3, DM4, and DS3 almost abrogated their signaling capability, indicating that they are non-functional receptors.

### Effects of variants on TGFβ-mediated growth regulation

**Figure 7:**
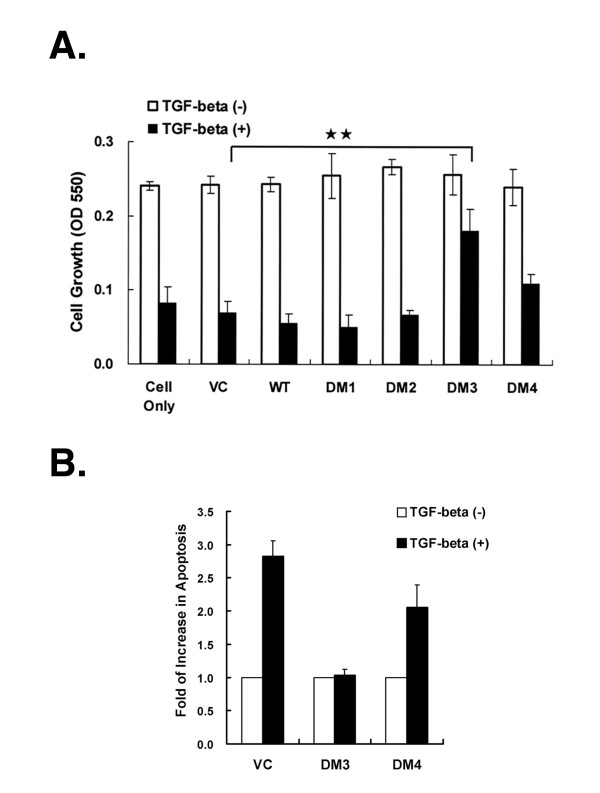
**Effects of DM3 on TGFβ-induced growth inhibition and cell apoptosis**. *Panel A: Cell growth*. The MCF-7 transfectants were seeded at a density of 5000 cells per well in 24-well plates followed by TGFβ1 treatment or not for 96 h. The cell counts for each transfectants were quantified by a standard MTT assay. The results are presented as the means ± SEM of three independent experiments. ** Indicates that the DM3 variant rescued the cells from TGFβ-induced growth suppression (*P *< 0.01). *Panel B: TGFβ-induced cell apoptosis*. Transfectants (2 × 10^5 ^cells) expressing DM3, DM4, or vector control (VC) were seeded and stimulated with (+) or without (-) 5 ng/mL of TGFβ1 for 72 h. The cells were collected and stained with Annexin V and propidium iodide for detection of apoptotic cells. The percentage of the apoptotic cells in the total cell population was determined by flow cytometry. Data are presented as the fold increases in cell apoptosis compared with those of corresponding untreated cells.

To test if the coexistence of these variants might alter the cellular function of TGFβ-sensitive cells, MCF-7 stable transfectants expressing individual TβRI variants were established and cell growth was determined by MTT assay. To eliminate possible artifacts associated with individual cell clones, mixed transfectants were used. No measurable difference on cell growth was observed among them in the absence of TGFβ. However, the DM3 variant, surprisingly, rescued 44.2% of growth suppression induced by TGFβ, but only partial prevention was seen in the cells expressing DM4 (Fig. 7A). The action of DM3 is, at least partially, attributed to the abolishment of TGFβ-induced apoptotic event (Fig. 7B). On the other hand, as expected for DM1 and DM2, expression of either variant only produced a slight difference from the control in the presence of TGFβ. This may result from two possibilities. First, MCF-7 cells have endogenous TGFβ receptors and intact downstream signaling pathways to respond to TGFβ stimulation. Increasing expression of exogenous signaling molecule(s) is not always proportional to enhance the signaling magnitude or biological outcomes [12,14]. Second, it might be caused by saturated signaling capacity in the cells. Furthermore, the expression level of DM1 or DM2 is quite low and probably barely affects endogenous signaling in TGFβ-responsive MCF-7 cells. In summary, these results suggest that the existence of some TβRI variants in cancer cells can bestow them with a growth advantage in a tumor microenvironment such as that in malignant pleural effusions with high levels of TGFβ.

## Discussion

The type I receptor of TGFβ (TβRI) has a central role to transmit TGFβ stimulation into the nucleus by activating its downstream Smad- and non-Smad-mediated pathways and modulating gene transcriptions to conduct its biological effects in cells [15,16]. Functional disruption, mutation, or aberrant expression of the *Tgfbr1 *gene could cause many human diseases, including cancers [3-6]. In the present study, we identified two additional functional elements in the signal peptide of TβRI beside published mutations in the 9A stretch. Sequence loss or deletion of a 12-residue peptide upstream to the 9A stretch, which is encoded by nucleotides 16–51, showed a dramatic impairment of TβRI expression because of its labile protein stability. On the other hand, a 13-residue peptide immediately downstream to the stretch is apparently involved in receptor-mediated signaling activation. In contrast, removal of the 9A stretch itself had only a mild effect on the protein expression and function of TβRI. Intriguingly, Pasche *et al*. recently reported that deletion of three alanine residues in the stretch conferred certain advantages for tumor growth and was associated with the malignancy of colorectal cancer [13]. Our study also demonstrated that the presence of the DM3 variant could counteract TGFβ action. These findings disclose an unexpected additional complexity in the response of tumor cells to TGFβ-mediated growth regulation.

In contrast to previously reported TβRI variants, the four newly discovered truncated forms defined in this study are apparently not caused by somatic mutations. Germ-line predisposing or cancer-associated somatically acquired mutations are usually considered the major cause for malfunction of the *Tgfbr1 *gene. Those mutations are composed of nucleotide transversion, insertion, and deletion mutations. Around 30% of patients with ovarian cancer bear a dinucleotide insertion in exon 5 of the *Tgfbr1 *gene [9]. Another tumor-susceptible allele is *Tgfbr1-6A*. A meta-analysis of 12 case-control studies by Pasche *et al*. [4] indicates that *Tgfbr1-6A *carriers have an increased risk of colon, breast, and ovarian cancers compared with non-carriers. In addition, there was a much higher frequency of this allele found in colorectal tumor tissue than in germ-line DNA extracted from the same patients [13], suggesting that acquisition of *Tgfbr1-6A *allele may be contributed by cancer-associated somatic deletions. However, this clinical correlation between the frequency of this allele and increased cancer risk is quite controversial since other studies did not reach the same conclusion [17-19]. One possible interpretation for this is that *Tgfbr1-6A *is also a common polymorphism in healthy individuals (11.2% to 19.7%) [11]. Hence, the frequency between cancer patients and controls is not distinctly different. A meta-analysis of case-control studies for heterogeneity from different groups may be marginally significant. Intriguingly, neither our laboratory nor others [20] have found this mutant allele in patients with lung cancer. Hence, acquisition of lung cancer-associated mutations to TβRI may be through post-transcriptional processes rather than because of sequence defects in its genomic DNA. One plausible mechanism for this is RNA editing, as several mammalian tumor-associated transcript mutants can be generated through this process [21-23].

RNA editing is a widespread phenomenon in eukaryotes and it involves insertions, deletions, or nucleotide conversion leading to post-transcriptional base changes in mRNA and tRNA [24]. This cellular process is also observed in mammalian transcripts and some of them are associated with tumorigenesis such as in the glutamate receptor [21], *Ptpn6 *[22], and *Wt-1 *[23]. Consistent with our finding, Choi [10] also reported an insertion of four or five amino acid residues in the carboxyl terminus of extracellular domain of naturally occurring rat *Alk-5 *(*Tgfbr1*) transcripts. This finding further strengthens our hypothesis that cancer cells may modulate their sensitivity to TGFβ by generation of its receptor variants through a post-transcriptional process and interfere with endogenous existing signaling. At least, DM3 in this study may be such a case.

Sequence deletions in the *Tgfbr1 *transcripts are not merely through a single biological process. In addition to the truncations in the signal peptide, the deletion of nucleotides 344–574 in the sequence for DM3 and nucleotides 97–972 in that for DM4 are consequences of major alternative gene splicing. Alignment of the published human *Tgfbr1 *gene sequence [25] with the nucleotide sequences encoding these two variants revealed that the deletions occur precisely between exons 2 and 4 for DM3 and between exons 1 and 6 for DM4. Hence, generation of these transcript variants is probably involved in at least two post-transcriptional processes: alternative RNA splicing or RNA editing or even a yet undefined biological process.

Regulation of protein degradation requires accurate identification. Frequently, a small region of protein sequence provides information that targets the protein for degradation such as the degradation motif of IκB [26], the stability-regulating region of MOS [27], and the N-terminal residues of some proteins [28,29]. The action for the degradation is either mediated by ubiquitin-dependent [30] or -independent [31] proteolytic pathways. Removal of the 12-residue peptide upstream to the 9A stretch in the DM1, DM2, and DS1 variants greatly shortened their protein half-lives (Fig. 5). Treatment with MG132, an ubiquitin-proteasome inhibitor, but not of lactacystin, a highly specific inhibitor of the 20S proteasome, rescued them from degradation. Therefore, this 12-residue peptide might have an indispensable role in the protection of TβRI against ubiquitin-dependent protein degradation. The protective role played by this peptide is probably to target a newly synthesized polypeptide of TβRI onto the endoplasmic reticulum membrane and prevents it from prolonged exposure to cytoplasmic proteolytic machinery. However, this is not the case for TGFBR1-6A. Pasche *et al*. [13] showed that the removal of three alanine residues from the 9A stretch did not measurably affect its membrane targeting. Thus, the essential role of the 12-residue peptide for membrane targeting of TβRI variants needs to be clarified.

Additionally, a region C-terminal to the 9A stretch in the signal peptide, ranging from residue 27 to residue 39, has a tremendous effect on TβRI-mediated transcriptional activation. Lack of this region in the DM3, DM4, and DS3 variants showed no dramatic difference from the wild-type one in their protein expression, but all failed to conduct TGFβ signaling (Fig. 6). This result clearly indicates the critical role of this region for the function of TβRI. Based on amino terminal sequencing in a previous report [13] or using a SignaIP program [32], the data indicate that the major cleavage site of the putative signal peptide is located between residues 33 and 34. Hence, loss of the cleavage site could be a potential factor accounting for DS3's refractoriness to TGFβ. One plausible speculation is that the signal peptides un-cleaved from these variants, and in turn, interfere with their ability to bind with ligand. However, there might be other residues in this region critical for the receptor function.

The co-existence of TβRI variants might confer survival benefits to tumor cells. This hypothesis is supported by our present data, showing that the expression of DM3 enabled MCF-7 cells to resist TGFβ-induced growth inhibition markedly. A similar observation was also reported in the TGFBR1-6A variant [13]. However, in contrast to this variant, the DM3 protein not only loses the truncated region of DS3, but also lacks the transmembrane domain, juxtamembrane region, and GS motif. In our gene reporter and cell proliferation assays in R1B cells (Fig. 6), those data further confirm that DM3 is a non-functional receptor and mainly localized in the cytosol. One previous study by Saitoh *et al*. [33] showed that a deletion mutant lacking its juxtamembrane region could be still associated with TβRII and transduce TGFβ signaling. Therefore, we speculate that this DM3 variant can modulate TGFβ-mediated signaling activation by competing with endogenous wild-type TβRI in the cells for association with the type II receptor and thereby blocking some downstream signaling pathways. This working hypothesis is currently supported by our unpublished data, showing that DM3 could significantly abolish TGFβ-induced JNK activation and have a potential role in tumorigenesis. However, another possibility that secondary signals generated by this variant may directly deregulate the TGFβ-induced cell apoptosis should also not be ruled out since it still contains an intact serine/threonine kinase domain. Compared to DM3, DM4 loses several kinase subdomains, particularly for subdomains III-V, which have been shown to include a key region for the transduction of TGFβ signaling [34]. The expression of this variant could also rescue TGFβ-induced growth suppression in a cell although the magnitude of the blocking effect was much weaker than that of DM3. If the molecular mechanism mediated by DM4 is the same as by DM3 remains to be elucidated.

Although the involvement of TGFBR1-6A and our newly discovered variants in tumorigenesis has not been substantially validated *in vitro *or *in vivo *studies, this implication seems to be supported by published data from several clinical studies. Thus, Chen *et al*. [8] performed genotyping for the *Tgfbr1 *gene in 34 patients with ovarian cancer. The data showed that 7 out of 34 (21%) patients had either deleted or point mutations in the truncated regions of DM3 or DM4. Wang, *et al*. [9] found that around 31.3% of ovarian cancer patients had a 2-nucleotide insertion at codons 276–277 (nucleotides 825–831), also located in this region. Thus, these findings suggest that mutations in *Tgfbr1 *are likely responsible for human ovarian carcinogenesis. However, the evidence from our and Zhang's [20] laboratories obviously indicate that mutations of *Tgfbr1 *probably do not result from defects in its DNA level. Hence, for association of *Tgfbr1 *mutations with lung cancer, a large-scale case-control study on mutations of its transcript is required to test any correlation between the frequency of mutated transcripts and their potential to cause malignancy.

## Conclusion

Loss of functional TβRI resulting in insensitivity to TGFβ is frequently associated with tumor malignancy [35,36]. The disclosure of these two hitherto undiscovered regions on the signal peptide of TβRI in this study adds pivotal knowledge to the molecular understanding of its protein expression and receptor function and provides an alternative molecular target for monitoring disease progression. First, these data demonstrate that a small deletion in the putative signal peptide of TβRI is a common feature for the pleural effusion-derived tumor cells we have examined so far, but this is not found in normal control cells. Second, the serial site-directed deletion mutagenesis resolved an indispensable role for TβRI protein stability of the 12-residue peptide upstream to the 9A stretch of the signal peptide. Deletion of a 13-residue peptide downstream to the 9A sequence produced serious impairment to receptor-mediated transcriptional activation. Third, study of the effects of these naturally occurring variants on cell growth has provided a plausible interpretation for how effusion tumor cells can survive in a microenvironment enriched with TGFβ. Finally, these observations indicate that a small sequence deletion in the signal peptide can cause distinct consequences for TβRI protein expression or receptor function and suggest that DM3 might act as an oncogene, regulating TGFβ-induced pathways that may be amenable to therapeutic intervention.

## Methods

### Cell culture

HEK 293 cells were cultured in Dulbecco's modified Eagle's medium (Invitrogen, Carlsbad, CA) supplemented with 10% fetal bovine serum (FBS), penicillin (100 units/ml), and streptomycin (100 μg/ml). TβRI-deficient R1B, MCF-7, and TβRI variant-transfected cells were cultured in Minimum Essential Medium in the presence of 10% FBS, 10 mM sodium pyruvate, and 1 mM nonessential amino acids (Invitrogen). The CA427, CA528, and CA1109 purified primary effusion tumor cells were cultured in ACR-4 medium [37] supplemented with 10% FBS for one or two passages *in vitro*.

### Sample processing

Pleural effusions were collected by thoracocentesis from three patients with lung adenocarcinoma following the tenets of the Helsinki declaration under the auspices of the National Health Research Institutes (NHRI) Institutional Review Board. The fluids were centrifuged within 2 h at 300 × *g *for 10 min to pellet the effusion cells. Separation of tumor cells from effusion-associated lymphocytes was performed using serial gradient centrifugation with Ficoll and Percoll (Pharmacia, Uppsala, Sweden) as described previously [38]. The purity of tumor cells in the fraction, as determined by cytological examination, was between 80–90%.

### RT-PCR and gene cloning

Total RNA from WI38 cells, human umbilical endothelial cells (HUVECs), and effusion-derived tumor cells was extracted using the High Pure RNA Tissue kit (Roche Diagnostics, Indianapolis, IN) and reverse-transcribed using SuperScript III reverse transcriptase (Invitrogen) as described in the manufacturer's instructions. Subsequently, nested-PCR reactions were performed to clone a *Tgfbr1 *coding sequence containing AUG start and UAA stop codons using two pairs of gene-specific primers, P1 and P2 (Fig. 1C), located in the 5' – and 3' -untranslated regions of the *Tgfbr1 *gene (NCBI database: NM_004612). To facilitate gene amplification of the highly GC-rich TβRI sequences, Advantage-GC polymerase (Clontech Co., Mountain View, CA) was used according to the manufacturer's instructions. Subsequently, PCR products were cloned into pIVEX2.5 (Roche) to generate C-terminus HA-tagged wild-type TβRI and its variants. Subsequently, the gene inserts were subcloned into the pcDNA3.1 vector (Invitrogen) at the 5' -*Hind* III and 3' -*Apa *I sites to improve protein expression in HEK 293 cells.

To generate mutants with truncations in the signal peptide, site-directed deletion mutagenesis reactions were carried out using designed primers (Table 2) and priming wild-type *Tgfbr1 *gene to truncate nucleotides 16–51 (DS1), 52–78 (DS2), or 79–117 (DS3), as described previously [39].

**Table 2:** List of primers used for reverse transcriptase-polymer chain reactions, site-directed deletion mutagenesis, and ribonuclease protection assay

**Gene**			**Sequence**
RT-PCR			
***TβRI***	**P1**	S:	5'-CGAGGTTTGCTGGGGTGAGGCA-3'
		AS:	5'-CTCAGTGAGGTAGAACAACTGACC-3'
	**P2**	S:	5'-AACAAGCTTTCCACCATGGAGGCGGCGGT-3'
		AS:	5'-GTTCATATCTTACATTTTGATGCCTTCCTGT-3'
***β-Actin***		S:	5'-CCCTTTTTGTCCCCCAAC-3'
		AS:	5'-CTGGTCTCAAGTCAGTGTACAGGT-3'
***TβRI-HA***	**P3**	S:	5'-ATCTATGCAATGGGCTT-3'
		AS:	5'-AGCGTAATCTGGAACATCGTA-3'
**Mutagenesis**			
***TβRI***	**DS1**	S:	5'-ATGGAGGCGGCGGTCGCGGCGGCGGCGGCGGCG-3'
		AS:	5'-CGCCGCCGCCGCCGCCGCGACCGCCGCCTCCAT-3'
	**DS2**	S:	5'-CTGCTCCTCCTCGTGCTGCTGCTCCCGGGGGCGACG-3'
		AS:	5'-CGTCGCCCCCGGGAGCAGCAGCACGAGGAGGAGCAG-3'
	**DS3**	S:	5'-GCGGCGGCGGCGGCGGCGCTCTGTACAAAAGACAAT-3'
		AS:	5'-ATTGTCTTTTGTACAGAGCGCCGCCGCCGCCGCCGC-3'
**RPA**			
***TβRI***	**DM1**	S:	5'-ACCATGGAGGCGGCGGT-3'
		AS:	5'-TAATACGACTCACTATAGGGACCACCACCTCCTTATTGC-AATGGTCCTGA-3'
	**DM2**	S:	5'-ACCATGGAGGCGGCGGT-3'
		AS:	5'-TAATACGACTCACTATAGGGACCACCACCTCCAGAGGGT-GCACATACAA-3'
	**DM3**	S:	5'-AACAAGCTTTCCACCATGGAGGCGGCGGTCGC-3'
		AS:	5'-TAATACGACTCACTATAGGGACCACCACCTCCTTCAGCT-ATACACATGCT-3'
	**DM4**	S:	5'-AACAAGCTTTCCACCATGGAGGCGGCGGTCGC-3'
		AS:	5'-TAATACGACTCACTATAGGGACCACCACCTCCATCTCTA-TGAGCAATGGCT-3'

S, sense primer; AS, antisense primer; RPA, ribonuclease protection assay

For quantification of *Tgfbr1 *transcript variants in the transfected HEK293 or R1B cells, reverse transcriptase-PCR (RT-PCR) reactions were performed using a pair of primer, P3, as indicated in Figure 3, aligned with 3'-coding sequence of *Tgfbr1 *and the HA tag sequence.*β-Actin *was acted as an internal control for those reactions.

### Western blotting

HA-tagged TβRI variants and β-Actin were analyzed by Western blotting. HEK 293 or R1B cells were transiently transfected with each gene construct using Lipofectamine (Invitrogen) or ExGene500 (Fermantas, Hanover, MD), respectively. For increasing protein expression, transfected R1B cells were infected with T7 vaccinia viruses as described previously [40]. The transfectants were lysed 24 h after the transfection in a lysis buffer [10 mM Na_2_HPO_4_, 150 mM NaCl, 1% Triton X-100, 0.5% sodium deoxycholate, 0.1% SDS, 10 mM NaF] supplemented with 1 × protease inhibitor cocktail (Roche Diagnostics). Approximately 15 or 150 μg of the lysates were resolved by electrophoresis in 10% or 15% SDS-containing polyacrylamide gels, as indicated in the figure legends. The proteins were blotted onto nitrocellulose transfer membranes (Schleicher & Schuell, Keene, NH) and probed with anti-HA-tag (Covance, Berkeley, CA), or anti-β-Actin (Sigma, St. Louis, MO) antibodies. The immuno-complexes were detected by probing with anti-mouse or -rabbit IgG conjugated with HRP (Jackson ImmunoResearch, Cambridgeshire, UK) and visualized using the SuperSignal chemiluminescence detection system (Pierce, Rockford, IL).

### Reporter gene assay

Receptor-mediated transcriptional activation of wild-type TβRI and its truncated variants was determined by measuring TGFβ-induced luciferase activity. Each gene construct or a control plasmid was co-transfected into R1B cells with the p3TP-Lux reporter gene using ExGene 500 (Fermentas). To normalize the transfection efficiency, 0.05 μg of the Renilla luciferase reporter plasmid pRL-TK (Promega, Madison, WI) was also utilized in the experiments. After 24 h, the transfectants were starved for 6 h by changing for fresh media supplemented with 0.2% FBS, and then treated with 5 ng/ml TGFβ1 (R&D systems, Minneapolis, MN) for 24 h. Firefly and Renilla luciferase activities in each transfected cell lysate were detected using Dual-luciferase reporter system (Promega) in a TD-20/20 luminometer (Turner Designs, Sunnyvale, CA).

### Flow cytometric analysis

Examination of transfection efficiency with different gene constructs was performed by co-transfection with the pEGFP plasmid encoding green fluorescent protein. One microgram of each gene construct and 0.1 μg of pEGFP were transfected into 3–5 × 10^5 ^cells and incubated for 24 h. Flow cytometric analyses were used to determine the percentage of green fluorescence-positive cells in total cell population using a FACSCalibur machine (BD Bioscience, San Jose, CA).

For analysis of TGFβ-induced cell apoptosis, 2 × 10^5 ^stable-transfectants expressing the DM3, or DM4 variants were seeded onto each well of 6-well plates and cultured overnight at 37°C. After TGFβ1 treatment for 72 h, cells were collected, washed with PBS twice, and stained with Annexin V/propidium iodide (PI) according to manufacturer's instructions (BioVision, Mountain View, CA). The results are presented as fold of increase in cell apoptosis as compared with corresponding untreated cells.

### Ribonuclease protection assay

The existence of *Tgfbr1 *naturally occurring transcript variants was confirmed by RNase protection analysis using the RPA III kit (Ambion, Austin, TX). PCR reactions using primers listed in Table 2 generated variant-specific amplicons with a 12-nt linker and a T7 promoter sequences located in the 3' end of the amplicons. ^32^P-labeled antisense riboprobes were prepared using a T7 transcription kit (Ambion) to yield 275, 204, 163, and 102 nt probes for DM1, DM2, DM3, and DM4, respectively. Total RNA was extracted from CA1109 effusion tumor cells. Ten μg of the RNA aliquot was hybridized with individual or mixed ^32^P-labeled antisense probe (5 × 10^4 ^cpm) for 16 -18 h at 42°C, digested with RNase A/T1, and resolved on a 6% acrylamide sequencing gel containing 8 M urea. The protected RNA fragments were visualized by autoradiography.

### In vitro transcription/translation

A plasmid (1 μg) coding for wild-type TβRI, DM1 variant, or luciferase control protein was transcribed and translated in a coupled reticulocyte lysate system (Promega). To visualize the translated proteins, [^35^S] methionine (1175 Ci/mmol) (PerkinElmer, Boston, MA) was used. The radiolabeled proteins were resolved in a 10% SDS-PAGE and autoradiographed with X-ray film.

### Cell growth assay

Stably transfected MCF-7 cells (5 × 10^3^) expressing individual variants, or vector control cells were seeded and treated with TGFβ1 for 96 h or not. The cell proliferation was determined by incubating the cells with 200 μl of fresh medium containing 1 mg/ml 3-(4,5-dimethylthiazol-2-yl)- 2,5-diphenyl tetrazolium bromide (MTT) (Sigma-Aldrich) for 4 h. After removal of the MTT solution, the resulting formazan crystals were dissolved completely in an ethanol/DMSO mixture (1:1), transferred to the wells of 96-well plates, and quantified using an ELISA plate reader (Metertech 960, Metertech Inc, Taipei, Taiwan) with the absorbance measured at 550 nm. Triplicate wells were assayed for each experiment and three or four independent experiments were performed, as indicated in the figure legends. Data is expressed as the mean of OD_550 _± standard deviation.

## Abbreviations

TGFβ, transforming growth factor-beta; TβRI, TGFβ type I receptor; 9 A stretch, 9 alanine stretch; TGFBR1-6A, TGFβ type I receptor with 3 alanine deletion; MTT, 3-(4,5-dimethylthiazol-2-yl)-2,5-diphenyl tetrazolium bromide; HUVEC, human umbilical endothelial cell; TPA: 12-tetradecanoylphorbol-13-acetate; HRP, horseradish peroxidase; PAI-1: plasminogen activator inhibitor 1

## Authors' contributions

KLC, WHL, YYY, and SJCL performed experiments and analyses. NYS conceived of the study and contributed to the writing of the manuscript. All authors read and approved the final manuscript.
